# Combined hepatocellular-cholangiocarcinoma: from genesis to molecular pathways and therapeutic strategies

**DOI:** 10.1007/s00432-024-05781-8

**Published:** 2024-05-23

**Authors:** Simona Gurzu, Rita Szodorai, Ioan Jung, Laura Banias

**Affiliations:** 1 Department of Pathology, Pharmacy, Science and Technology, George Emil Palade University of Medicine, 38 Gheorghe Marinescu Street, 540139 Targu Mures, Romania; 2 Research Center of Oncopathology and Transdisciplinary Research (CCOMT), Targu Mures, Romania; 3 Romanian Academy of Medical Sciences, Bucharest, Romania

**Keywords:** Stem cells, Hepatocellular carcinoma, Cholangiocarcinoma, Carcinosarcoma, Lymph node metastases, Primary liver cancer

## Abstract

Hepatocellular carcinoma (HCC) and intrahepatic cholangiocarcinoma (ICC) are the most common primary liver cancers. Little is known about the combined hepatocellular-cholangiocarcinoma (cHCC-ICC) variant and the proper therapeutic strategies. Out of over 1200 available studies about cHCC-ICC, we selected the most representative ones that reflected updated information with application to individualized therapy. Based on literature data and own experience, we hypothesize that two molecular groups of cHCC-ICC can be identified. The proposed division might have a significant therapeutic role. Most cases develop, like HCC, on a background of cirrhosis and hepatitis and share characteristics with HCC; thus, they are named HCC-type cHCC-ICC and therapeutic strategies might be like those for HCC. This review also highlights a new carcinogenic perspective and identifies, based on literature data and the own experience, a second variant of cHCC-ICC called ICC-type cHCC-ICC. Contrary to HCC, these cases show a tendency for lymph node metastases and ICC components in the metastatic tissues. No guidelines have been established yet for such cases. Individualized therapy should be, however, oriented toward the immunoprofile of the primary tumor and metastatic cells, and different therapeutic strategies should be used in patients with HCC- versus ICC-type cHCC-ICC.

## Introduction

Despite improvements in diagnostic and therapeutic approaches, liver cancer remains the sixth most common malignancy worldwide and the third leading cause of cancer-related death after colorectal and lung cancer (Gurzu et al. 2021; Turdean et al. 2012; Roßner et al. 2023). Over one-third of primary malignancies of the liver are hepatocellular carcinomas (HCCs) (Gurzu et al. 2021; Turdean et al. 2012). The second most common histopathological subtype is intrahepatic cholangiocarcinoma (ICC), which represents 6–15% of all primary malignant tumors of the liver (Turdean et al. 2012; Roßner et al. 2023). Other infrequent primary malignant tumors include hepatoblastoma, angiosarcoma, and bile duct cystadenocarcinoma (Turdean et al. 2012).

In 2–5% of patients, HCC and ICC can occur synchronously as two independent tumors in different segments of the liver (Jung et al. 2017) or as a mixed tumor with features of both HCC and ICC, which is known as combined hepatocellular-cholangiocarcinoma (cHCC-ICC) (Turdean et al. 2012; Roßner et al. 2023; Garancini et al. 2014; Lee et al. 2006). Synonyms for this condition include “combined hepatocellular carcinoma and cholangiocarcinoma,” “mixed hepatocellular-cholangiocarcinoma,” “biphenotypic hepatobiliary primary liver carcinoma,” “HCC with dual hepatocellular-biliary phenotype,” “mixed hepatobiliary carcinoma,” and “hepatocholangiocarcinoma” (Brunt et al. 2015; Sempoux et al. 2019).

This review aimed to provide an update on the literature regarding cHCC-ICC, an unusual and rare malignant tumor of the liver, whose carcinogenesis, diagnosis, and therapy are far from being well understood. Most previously published data are based on single-center retrospective analyses of small cohorts. This is the first review focusing on the unusual metastatic spread in the lymph nodes, a behavior that is contrary to HCC, and the predictive value of this parameter. As the therapeutic approach for cHCC-ICC is like that used for HCC, understanding their metastatic route, from genesis to clinical evolution, might change the oncologic management of such cases. No international guidelines are currently available for the therapeutic management of cHCC-ICC (Azizi et al. 2020).

**Fig. 1:**
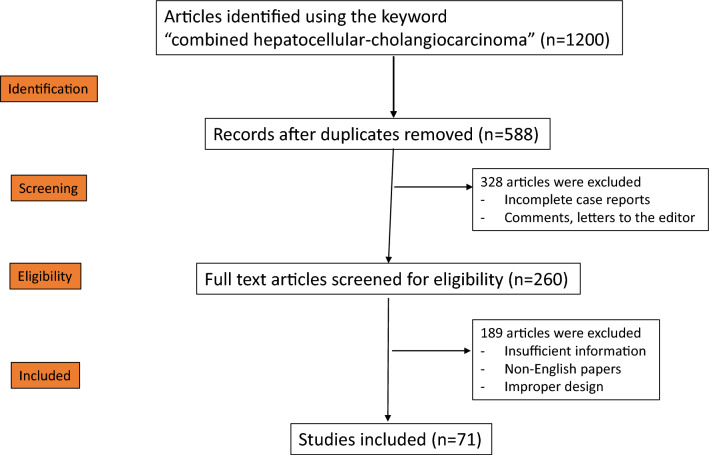
Preferred reported items for systematic reviews and meta-analyses (PRISMA) flow diagram for searching the PubMed and Web of Science databases between 1903 and 2024

To prepare this narrative review, the literature was systematically searched to identify representative papers focused on cHCC-ICC, from experimental to clinical studies. A comprehensive literature search of papers indexed in the Medline (PubMed) and Web of Science databases up to May 2024 was performed. The general search terms included “combined hepatocellular-cholangiocarcinoma,” “sarcomatous hepatocellular carcinoma,” “liver stem cells,” “liver stemness,” and “hepatocarcinogenesis”. The other MeSH terms and words searched included “liver cancer AND lymph node metastases,” “synchronous AND hepatocellular,” and “synchronous and cholangiocarcinoma.” Data assessment was conducted independently by the four authors (GS, SR, JI, and BL) using predefined terms (Fig. 1).

## Historical data, epidemiology, and clinical features

The uncommon primary liver neoplasm cHCC-ICC was first described by Wells as a primary carcinoma of the liver, without significant data to be considered further (Wells 1903). In 1949, Allen and Lisa first classified cHCC-ICC based on five cases (Allen and Lisa 1949). It was called “combined liver cell and bile duct carcinoma” and defined by three histologic variants: distinct limits between the two components (Allen type A), separate nodules of HCC and ICC in the same patient (Allen type B), and a single mass showing an intimate interfusion between HCC and bile duct carcinoma (Allen type C) (Allen and Lisa 1949; Jarnagin et al. 2002; Wachtel et al. 2008; Lee et al. 2013).

Then, in 1959, Steiner called the tumor “cholangiolocellular carcinoma of the liver” based on 11 cases (Steiner and Higgison 1959). In 1985, Goodman et al., based on 24 other cases, first used the term CHCC-ICC and re-categorized it into three histologic variants: type I or “collision tumors”—incidental diagnosis of HCC and ICC separately in the same liver (Allen type B); type II or “transitional tumors”—one single tumor with a progressive transition between HCC and ICC areas (Allen type A + C); and type III or “fibrolamellar tumors”—intermingled areas with histologic similarities to the fibrolamellar variant of HCC but with mucin-producing foci or pseudoglands (Brunt et al. 2015; Yin et al. 2012; Goodman et al. 1985).

These classifications were used until 2004, and synchronous HCC-ICC was long considered a variant of cHCC-ICC. About 500 cases of cHCC-ICC were reported between 1903 and 2004 (Brunt et al. 2015). Thereafter, the World Health Organization (WHO) recommended a re-classification, and today, synchronous HCC-ICC, collision tumors, and the combined variant that is called cHCC-ICC are recognized as separate entities (Garancini et al. 2014; Brunt et al. 2015). Only cHCC-ICC, which was first classified as Allen type C, is now considered a true cHCC-ICC (Lee et al. 2013).

Various incidences have been reported for cHCC-ICC, from a range of 0.4–6.3% of all primary hepatic tumors in Asia to 14.2% in Western countries (Garancini et al. 2014; Lee et al. 2006, 2013; Jarnagin et al. 2002; Wachtel et al. 2008). In cohorts of over 20,000 patients diagnosed with primary malignant liver tumors and followed up over a period of 30 years, cHCC-ICC accounted for 0.8–1% of all primary liver cancers (Garancini et al. 2014; Lee et al. 2006; Jarnagin et al. 2002; Wachtel et al. 2008; Yin et al. 2012).

The epidemiological data regarding cHCC-ICC are mostly provided by the Surveillance, Epidemiology and End Results (SEER) database. An analysis of data from 1988 to 2009 included 465 patients with cHCC-ICC from the US whose characteristics were compared with those of over 50,000 patients with HCC and 7181 with ICC (Garancini et al. 2014). This tumor was seen to be more frequent in white men over the age of 65 years. Later data showed that many patients had a history of cirrhosis (8–67%), chronic hepatitis B (9–70%), hepatitis C (1–17%), combined hepatitis B and C (6%), or alcohol abuse (7%) (Lee et al. 2006, 2013; Yin et al. 2012; Li et al. 2016; Wu et al. 2016; Zhao et al. 2016). Over 60% of cHCC-ICCs develop from chronic liver disease, with the parenchyma showing hepatocytic injuries (Li et al. 2016; Zhao et al. 2016) (Table 1).

**Table 1:** Demographic and clinical parameters of patients with pure hepatocellular carcinoma (HCC), pure cholangiocarcinoma (ICC), and combined hepatocellular-cholangiocarcinoma (cHCC-ICC) based on a review of literature data reported in USA, Asia and EU (Garancini et al. 2014; Lee et al. 2006, 2013; Yin et al. 2012; Li et al. 2016; Zhao et al. 2016; Chen et al. 2021; Gigante et al. 2023)

Parameter	cHCC-ICC	HCC	ICC
Male: female ratio	US—2:1 Asia—2–8:1	US—3:1 Asia—3–6:1	US—1:1 Asia—1–2:1
Median age at diagnosis (years)	US—62–63 Asia—50–55 (range 27–76 years)	US—63–65 Asia—50–58 (range 10–88 years)	US—68–70 Asia—56–62 (range 31–79 years)
Body mass index ≥ 30 kg/m^2^	Taiwan—20%	Taiwan—10%	Taiwan—9%
Hepatitis B virus infection	Asia—47%	Asia—41%	Asia—20%
Hepatitis C virus infection	Asia—15%	Asia—27%	Asia—8%
Hepatitis B and C	Asia—11%	Asia—14%	Asia—3%
Liver cirrhosis	Asia—50–67%	Asia—60–86%	Asia—20–25%
Race—US	75% whites	66% whites	80% whites
Reported prevalence—US			
1988–1995	14%	12%	18%
1996–2002	24%	28%	32%
2003–2009	62%	60%	50%
Therapy—TACE	Asia—43%	Asia—53%	Asia—30%
Therapy—Sorafenib	Asia—96%	Asia—96%	Asia—99%
5-year overall survival	US—11% Asia—24–36%	US—12% Asia—41%	US—6% Asia—22%
Disease free survival after hepatic resection	US—18% Asia—23–25%	US—21% Asia—18–68%	US—12% Asia—11–16%
Median progression free survival (months)	Asia—8–10 EU—7.5	Asia—23–25	Asia—5–7
Median overall survival (months)	Asia—15–47 EU—17	Asia—29–72	Asia—10–22

TACE-transarterial chemoembolization

Data from USA and EU were not reported in representative cohorts

Over 75% of patients with cHCC-ICC present lymph node involvement (Garancini et al. 2014), the rate of lymphatic spread being even higher than in patients with ICC and significantly lower than in those with HCC (Lee et al. 2006, 2013; Yin et al. 2012) (Table 2).

**Table 2:** Pathological features of pure hepatocellular carcinoma (HCC), pure cholangiocarcinoma (ICC), and combined hepatocellular-cholangiocarcinoma (cHCC-ICC) based on a review of literature data reported in EU, USA, and Asia (Gurzu et al. 2021; Garancini et al. 2014; Lee et al. 2006, 2013; Yin et al. 2012; Li et al. 2016; Zhao et al. 2016; Chen et al. 2021)

Parameter	cHCC-ICC	HCC	ICC
Vascular invasion	Asia—23–50%	Asia—2–32%	Asia—12–42%
Lymph node metastases	US—75% Asia—10–76%	Asia—0.06–2 maximum 30%	Asia—20–69%
Tumor size			
Median size (cm)	US—5–6 Asia—4–6	US—6–7 Asia—5–6	US—6–7 Asia—5–6
< 5 cm	US—54% Asia—39–53%	US—50% Asia—39–62%	US—43% Asia—21–53%
5.1–10 cm	US—35% Asia—23–33%	US—34% Asia—16%	US-42% Asia—28%
> 5 cm	US—46% Asia—51%	US—50% Asia—38%	US—57% Asia—37%
> 10 cm	US—11% Asia—13%	US—16% Asia—11%	US-15% Asia—11%
Tumor number			
Single	Asia—51–84%	EU—20% Asia—52–80%	Asia—31–90%
Multiple	Asia—16–27%	EU-80% Asia—20–31%	Asia—10–31%
Tumor stage			
I–II	Asia—63%	Asia—58%	Asia—28%
III	Asia—18%	Asia—31%	Asia—22%
IV	Asia—19%	Asia—11%	Asia—51%
Capsule formation	Asia—27–30%	Asia—52–60%	Asia—19–28%

Data from US and EU were not reported in representative cohorts

Pre-operatively distinguishing between HCC, ICC, and the mixed variants, based on the serum values of alpha-fetoprotein (AFP) and CA 19-9 is often difficult (Table 3). AFP is considered more specific for HCC while CA19-9 is more sensitive for the diagnosis of ICC (Lee et al. 2006; Yin et al. 2012; Zhao et al. 2016). Simultaneously increased serum values of both AFP and CA19-9 can be detected in few than 10% of patients with pure HCC or pure ICC, 29–45% of those with synchronous HCC-ICC, and one third of patients with cHCC-ICC (Lee et al. 2006; Zhao et al. 2016; Zhou et al. 2008; Cao et al. 2013). In patients with cHCC-ICC, carcinoembryonic antigen (CEA) was reported to be elevated in 17–29% of cases, similar to the patients with ICC (Garancini et al. 2014; Lee et al. 2006; Yin et al. 2012; Zhao et al. 2016; Zhang et al. 2024).

**Table 3:** Tumor markers, immunohistochemical and molecular profile of pure hepatocellular carcinoma (HCC), pure cholangiocarcinoma (ICC), and combined hepatocellular-cholangiocarcinoma (cHCC-ICC) based on a review of literature data (Lee et al. 2006; Sempoux et al. 2019; Yin et al. 2012; Zhao et al. 2016; Zhou et al. 2008; Cao et al. 2013; Zhang et al. 2024; Maximin et al. 2014; Xue et al. 2019; Rizell et al. 2020)

Parameter	cHCC-ICC	HCC	ICC
Elevated serum biomarkers			
Alpha fetoprotein (AFP)	21–70%	32–66%	9–30%
CA 19-9	21–83%	2–10%	44–58%
CA 19-9 + AFP	20–30%	2–9%	4–10%
CEA	17–30%	2%	18–25%
Mutated genes			
TP53	49–80%	31%	22%
pTERT	23–80%	46%	Absent
CTNNB1	6–54%	16–27%	Absent
Immunohistochemical markers	CEA, HepPar1, AFP, glypican 3, cytokeratin 7/19, epithelial membrane antigen (EMA), nestin, CD10, MUC1	HepPar1, AFP, arginase 1, glypican 3, nestin	Cytokeratin 7/19, EMA, EpCAM, CEA

From an imaging perspective, HCC is regarded as a “fast wash-in and fast wash-out” tumor, with early arterial hyperenhancement and early washout (Azizi et al. 2020; Wu et al. 2016). Pure ICC in dynamic computer tomography (CT) or MRI shows a peripherally enhanced area in the early phase, followed by progressive centripetal enhancement and bile dilatation (Azizi et al. 2020). This delayed enhancement and delayed washout is known as “delayed reinforcement” (Wu et al. 2016; Horvat et al. 2018) and is explained by the fibrous stroma that does not allow early absorption of the contrasting substance (Maximin et al. 2014).

In CT scans, cHCC-ICC predominantly appears as a single hepatic hypoechoic or isodense nodule less than 5 cm in size, but its imaging features overlap with those of HCC and ICC (Sempoux et al. 2019; Azizi et al. 2020; Li et al. 2016). Over 65% of cHCC-ICCs develop in the right lobe of the liver (Li et al. 2016). Typical ICC may be exhibited in cases that histologically show an ICC component in over 50% of the tumor, while the other cases present characteristics of HCC in imaging studies (Wu et al. 2016). The enhancing pattern does not depend only on the proportion of HCC versus ICC but also on the type of stroma. A cHCC-ICC with scirrhous stroma will show delayed enhancement in comparison with medullary-type cHCC-ICC (Maximin et al. 2014).

The MRI features of cHCC-ICC were examined in fewer than 10 studies published up to 2014 (Maximin et al. 2014), without a significant increase in later years. In MRI examinations, cHCC-ICC may appear as a hypointense mass with/without a central hypointense focus corresponding to a central fibrous or ICC area (Maximin et al. 2014). Ultrasound examination reveals a round hypoechoic mass whose heterogeneity might reflect a mixed histologic variant (Maximin et al. 2014).

## Histology, tumor stage, and immunoprofile of cHCC-ICC versus HCC and ICC

### HCC

The differential diagnosis of cHCC-ICC first considers pure HCC and pure ICC. HCC can show a solid, trabecular, or pseudo-glandular pattern, with commonly seen vascular invasions and multicentricity. This phenomenon is called satellitosis. Bile production, rich eosinophilic cytoplasm of polygonal cells, prominent nucleoli, and hyaline bodies are also characteristic of HCC (Lee et al. 2006; Yin et al. 2012). Over 60% of HCCs are differentiated tumors (G1–G2) (Lee et al. 2006; Yin et al. 2012). Cirrhosis is frequently seen in peri-tumoral parenchyma.

The immunohistochemical (IHC) profile of HCC (Table 3) is based on cytoplasmatic positivity for hepatocyte paraffin 1 (HepPar1) and other markers such as AFP, arginase 1, and glypican 3. Canalicular and sinusoidal staining for CD10, CD34, and pCEA (polyclonal CEA), along with reticulin framework disintegration, is also characteristic of HCC, as is nuclear positivity for hepatocyte nuclear factor 4 alpha (HNF4a) (Brunt et al. 2015; Gurzu et al. 2015). The tumor cells do not express cytokeratins (CTKs) such AE1/AE3, CTK7, CTK13, CTK19, CTK20, or CDX2.

### ICC

Pure ICC can be macroscopically seen as a mass-forming tumor and infiltrative periductal or intraductal growth (Lee et al. 2006). It is histologically defined by glandular architecture and abundant fibrous stroma embedding irregular, large tubular structures with round vesicular nuclei, no prominent nucleoli, and infrequent mucin production (Lee et al. 2006; Yin et al. 2012). Over 60% of ICCs are differentiated tumors (G1–G2) (Lee et al. 2006; Zhao et al. 2016). The tumor cell cytoplasm is marked in 90% of cases by CTK7, CTK19, epithelial membrane antigen (EMA; Muc-1), epithelial adhesion molecule (EpCAM), and CEA (Table 3), which are indicators of bile duct differentiation, along with nuclear staining for transcription factors Sox-9 and Sox-10 (Lee et al. 2006; Brunt et al. 2015; Yin et al. 2012; Goodman et al. 1985; Vinay et al. 2023). ICC does not express HepPar1, arginase 1, CD10, AFP, or glypican 3 (Lee et al. 2006).

Some markers, such as CTK8/18, CEA, alpha-1 antitrypsin, fibrinogen, and Immunoglobulin G may be found in both HCC and ICC as well as in combined tumors and are not useful for differential diagnosis (Goodman et al. 1985).

### Classic cHCC-ICC

According to the WHO, classic cHCC-ICC is defined as the unequivocal architectural coexistence of both hepatocytic and cholangitic differentiation within the same tumor, irrespective of the percentage of each component (Brunt et al. 2015; Sempoux et al. 2019; Yin et al. 2012). Regarding histological differentiation, between 39% (Lee et al. 2006) and 80% of cHCC-ICCs (Yin et al. 2012; Zhao et al. 2016) are differentiated tumors (G1–G2). Most of the published studies included Asian patients from South Korea (Lee et al. 2006) and China (Yin et al. 2012; Zhao et al. 2016).

It is difficult, however, to diagnose cHCC-ICC microscopically without using a large panel of IHC antibodies. In typical/classic cHCC-ICC, the architectural features of HCC and ICC are clearly seen as distinct or intermingled areas with poorly defined or sharp transitions between the two components, without including collision tumors (Brunt et al. 2015; Sempoux et al. 2019). In these cases, a deep-learning-based reclassification was recently done by Calderaro et al. based on the percentage of HCC versus ICC components (Calderaro et al. 2023).

In other cases, the diagnosis of cHCC-ICC is exclusively based on the immunoprofile (Table 3), but the WHO does not recommend diagnosing it without supportive histomorphology (Sempoux et al. 2019). Pure HCC can show biliary findings via immunophenotyping, and pure ICC can express a hepatocytic immunoprofile (Brunt et al. 2015). AFP and nestin mark the HCC component in one-third and 67% of cases, respectively (Zhang et al. 2024). Half of cHCC-ICC cases also express CTKs (Goodman et al. 1985). Both components can also be marked simultaneously by hepatocyte and cholangiocyte markers (Zhao et al. 2016). Metastases show features of cHCC-ICC, pure HCC, or pure ICC (Sempoux et al. 2019; Zhao et al. 2016).

In the eighth edition of the American Joint Committee on Cancer (AJCC) system, the T1 primary liver cancers were reclassified based on a tumor size of 5 cm; similarly, reconsideration of the cHCC-ICC staging system is necessary (Satala et al. 2021; Deng et al. 2023). Recently, the use of the “tumor burden score” (TBS) was proposed for the more precise staging of cHCC-ICCs, especially multifocal ones. TBS is calculated based on the distance from the origin of a Cartesian plane and the formula TBS^2^ = (maximum tumor diameter)^2^ + (number of tumors)^2^ (Deng et al. 2023; Sasaki et al. 2018). A high TBS is associated with lower overall survival (OS) (Deng et al. 2023).

### cHCC-ICC with stem/progenitor cell phenotype and immunophenotype

A cHCC-ICC with stem cell features is characterized by the presence of small uniform cells with scant cytoplasm and inconspicuous nuclei in the transitional zones between the HCC and ICC components (Sempoux et al. 2019). These cells are called cancer stem cells, and their nuclei are marked by the fetal-type growth factor SALL-4, Oct4, Nanog, and Sox-9 (Brunt et al. 2015; Sempoux et al. 2019). Cytoplasmic positivity for progenitor cells such as CD117 (c-KIT), CD133, EpCAM, NCAM (CD56), EMA, nestin, CTK14, and CTK19, is also frequently seen (Brunt et al. 2015; Sempoux et al. 2019). This unusual variant of cHCC-ICC includes three subtypes: typical, intermediate, and cholangiocellular (Brunt et al. 2015). As per the last edition of the WHO classification, the use of this cHCC-ICC subtype is no longer recommended (Sempoux et al. 2019). Only intermediate cell carcinoma has been retained in the WHO classification, defined as having homogenous features intermediate between hepatocytes and cholangiocytes (Roßner et al. 2023; Sempoux et al. 2019; Azizi et al. 2020).

### Fibrolamellar cHCC-ICC

The fibrolamellar variant, described by Goodman et al. as type III cHCC-ICC, includes areas of fibrolamellar HCC and ICC. It occurs at younger ages, without an association with chronic liver disease, and has a slightly longer duration of survival compared with the other histological types (Brunt et al. 2015; Goodman et al. 1985). This variant was removed from the latest edition of the WHO classification (Sempoux et al. 2019).

### Sarcomatoid cHCC-ICC

In a small number of cases, besides the carcinomatous components with aspects suggesting hepatocellular and bile duct differentiation, sarcomatous elements can be present in variable percentages, increasing the difficulty of diagnosis. The sarcomatoid cHCC-ICC or hepatocellular-cholangiocellular sarcoma, also known as carcinosarcoma or hepatic sarcomatoid carcinoma, is another uncommon variant of cHCC-ICC, with specific imaging, histologic, and IHC features (Xiang et al. 2015; Gu et al. 2017). Its genesis is supposed to be related to the epithelial-mesenchymal transition (EMT) process undergone by aberrant liver progenitor cells (Xiang et al. 2015; Gu et al. 2017; Gurzu et al. 2019). Only 57 cases were published in the Medline database up to 2015 and other 63 between 2015 and 2024, most of them being reported after 2010 (Xiang et al. 2015).

The following case, diagnosed in our department (data not published previously), illustrates the characteristics of the sarcomatous variant of cHCC-ICC. An 87-year-old man presented to the Emergency Department complaining of non-specific symptoms such as pain in the right hypochondrium, nausea, vomiting, and lack of appetite. An abdominal CT scan showed multiple hepatic focal lesions with nonspecific loads, with diameters varying from 14 mm (third hepatic segment) to 103 mm (fourth hepatic segment), with one lesion compressing the right portal branch, which appeared to be thrombosed. Several adenopathies were also found in the hepatic hilum and periportal and peripancreatic regions. A blood analysis showed elevated bilirubin (direct bilirubin 6.13 mg/dL, total bilirubin 7.01 mg/dL) and continuously elevating liver enzymes, with the highest level of alanine transaminase (ALT) being 404 U/L and that of aspartate aminotransferase (AST) being 801 U/L. Under a microscope, the biopsy from hepatic tumoral tissue, reflecting a sarcomatoid cHCC-ICC, was composed of atypical large cells with abundant cytoplasm and marked nuclear pleomorphism, some with prominent nucleoli, arranged in solid areas. The spindle-shaped cells focally formed glandular structures, all placed in a desmoplastic stroma. Both the solid areas and the glandular components focally presented positive IHC expression of CTK AE1/AE3, CTK19, CTK18, and vimentin. Only the atypical ductal structures showed positive CTK7 expression. Membrane expression of β-catenin was observed, along with focal expression of S100 protein in the cytoplasm and nucleus. The proliferation index Ki-67 was below 50%. Immunostaining for Hepatocyte Specific Antigen (HSA), HMB45, CEA, AFP, CTK20, CD117, CD56, CTK8, the neuroendocrine markers synaptophysin and chromogranin, and CD105 were negative. Besides the characteristics described in this representative case, sarcomatous cHCC-ICCs can also comprise foci of osteosarcoma, chondrosarcoma, rhabdosarcoma, fibrosarcoma, or leiomyosarcoma (Xiang et al. 2015; Yoshuantari et al. 2023).

The positive IHC expression of S100, which was described in cHCC-ICC, is considered an indicator of poor histologic differentiation, aggressive behavior, and risk for distant dissemination. S100 enhances neoplastic cell migration by binding with proteins belonging to the cytoskeleton and with DNA binding factors. S100 proteins interact with p53 and change the composition of the extracellular matrix by influencing the function of matrix metalloproteinases. The S100A4 protein is related to the EMT and increases metastasizing capacity, S100A6 is involved in cell proliferation and invasion, and S100A8 and S100A9 seem to influence the initial steps in tumor formation (Maletzki et al. 2012; Chen et al. 2015; Li et al. 2014). In ductal adenocarcinoma of the pancreas. S100A6 enhances the EMT and cancer progression through β-catenin activation (Chen et al. 2015). An S100-positive sarcomatoid component was also described in a case of renal cell carcinoma that was initially misdiagnosed as a malignant peripheral nerve sheath tumor, with partial remission observed only after a change in therapy and a second surgical intervention (Miolo et al. 2014).

Sarcomatous cHCC-ICC should be differentiated from the newly described pseudoendocrine sarcoma, in which cells are marked by nuclear β-catenin (reflecting mutations of the *CTNNB1* gene), and show focal positivity for S100, desmin, and CD34, and negativity for CTKs (Papke et al. 2022).

## Carcinogenesis

It was long thought that HCC originates from hepatocytes and ICC from the duct epithelium (Garancini et al. 2014; Brunt et al. 2015). Then, it was hypothesized that due to tumor plasticity, HCC can be transformed into ICC and vice versa (Garancini et al. 2014; Zhao et al. 2016). This hypothesis is sustained by the cellular changes in the biliary duct that occur after liver injury when pseudo-canalicular spaces are formed surrounding the damaged hepatocytes and express biliary markers such as CTK7 and CTK19 (Brunt et al. 2015; Holczbauer et al. 2021). It is even thought that the cHCC-ICC originates from a pluripotent hepatic precursor cell (Lee et al. 2013; Tang et al. 2006) that is also called liver progenitor/stem cell or oval cell (Brunt et al. 2015; Sempoux et al. 2019; Calderaro et al. 2023; Zhou et al. 2010). This heterogeneous histological architecture was also considered as the result of “evolutionary cancer stem cells concept” (Oikawa 2016).

Human HCC lines proved that hepatocytes can be genetically programmed to undergo dual-directional differentiation and transform into bipotent cancer stem cells with hepatobiliary differentiation potential (Brunt et al. 2015; Zhao et al. 2016; Papke et al. 2022). However, the mechanism driving the trans-differentiation of hepatocytes into cholangiocytes and further ICC is far to be understood (Wang et al. 2018). It was experimentally proved to be related to specific molecular mechanisms such as the canonical Notch signaling pathway (Wang et al. 2018). Notch cascade is involved not only in the genesis of ICC from damaged hepatocytes but also influences the EMT phenomenon, genesis of pseuodocanalicular structures and even mesenchymal-to-epithelial or mesenchymal-to-endothelial transition, similar to other malignancies (Chen et al. 2020; Gurzu et al. 2013).

A pathomolecular study evaluating the loss of heterozygosity patterns in polymorphic microsatellite markers, in patients with cHCC-ICC versus those with pure HCC and ICC, showed the potential common origin of the two components of cHCC-ICC in the same clone (Zhao et al. 2016), which explains the intermingled, dually differentiated aspect of cHCC-ICC, with solid poorly differentiated areas expressing IHC markers for both HCC and for ICC. A supplementary feature is the simultaneous staining for CTK8/18 in normal hepatocytes, biliary ducts, and tumors with hepatoid or biliary differentiation. Another argument for the bilinear differentiation and high cellular plasticity is the constant positivity of cHCC-ICC cells for nestin, that is known as a marker for the bipotent progenitor oval cell (Zhang et al. 2024; Xue et al. 2019).

The clinical and therapeutic implications of the biphenotypic/hepatobiliary characteristics of cHCC-ICC are controversial. As discussed later in this review, cHCC-ICC is considered to have a biological potential that is intermediate between HCC and ICC but more like HCC. Consequently, most patients are still treated using the same protocols as for HCC (Chen et al. 2021), although the WHO includes cHCC-ICC in the group of hepatobiliary cancers (Sempoux et al. 2019).

An in-depth analysis of architecture and immunoprofile of cHCC-ICC, like the unusual lymph node metastatic predominance (Lee et al. 2013; Yin et al. 2012), leads us to consider cHCC-ICC as being a variant of ICC rather than HCC. This is supported by the identification of ICC components in the metastatic lymph nodes in some cases (Lee et al. 2011). The molecular homogeneity of cHCC-ICC, which is dissected in the next section of this review, supports the hypothesis that cHCC-ICC is molecularly closer to ICC than to HCC (Brunt et al. 2015) and might originate from the proliferated pseudo-ducts that surround damaged hepatocytes during chronic inflammation (Papke et al. 2022).

**Fig. 2:**
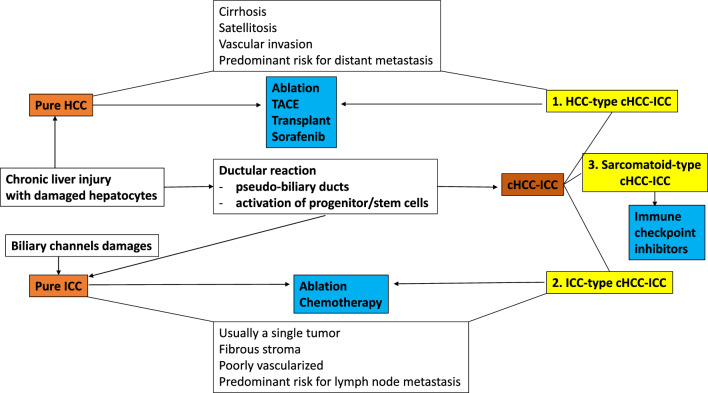
Cellular origins of hepatocellular carcinoma (HCC), intrahepatic cholangiocarcinoma (ICC), and combined hepatocellular-cholangiocarcinoma (cHCC-ICC) and their possible predictive value

Based on this theory, two variants of cHCC-ICC can be identified, with potential therapeutic implications (Fig. 2). The first variant is the HCC-type cHCC-ICC. Like HCC, this variant might be developed on a background of cirrhosis and hepatitis, it is well-vascularized but, although it might have a predominant trabecular architecture, in over 50% of the tumor area (Sheng et al. 2023), the tumor cells can express markers for both HCC and ICC and usually shows the molecular profile which typically occurs in HCC (Calderaro et al. 2023). This molecular variant presents, as compared with HCC, a lower density of CD8^+^ T cells and higher intensity of immune checkpoints, as indicators of immune escape properties of tumor cells (Xue et al. 2019) (Fig. 2).

The second variant can be named ICC-type cHCC-ICC. Its origins are rather related to the proliferated pseudo-ducts that surround damaged hepatocytes during chronic inflammation. These periportal progenitors are also called hybrid hepatocytes and are intermediates between hepatocytes and biliary tree stem cells (Oikawa 2016). The predominant component of ICC-type cHCC-ICC is more like ICC (Sheng et al. 2023), being characterized by a fibrotic stroma and higher risk for lymph nodes metastases. Similar to the HCC-type cHCC-ICCs, the tumor cells are biphenotypic and the molecular profile is more closely to ICC (Calderaro et al. 2023). The prognosis of HCC-type cHCC-ICCs seems to be better than those reported for ICC-type cHCC-ICC (Sheng et al. 2023).

Sarcomatous variant of cHCC-ICC is an extremely rare type which shows positivity for CTKs and vimentin and a more solid architecture.

## Molecular profile

Little is known about the pathomolecular profile of cHCC-ICC in comparison with HCC and ICC. Most studies emphasize the common molecular origin of the HCC and ICC components (Sempoux et al. 2019). Although the molecular profile of cHCC-ICC indicates an origin closer to ICC than HCC, gene mutations are not specific (Sempoux et al. 2019). The most common mutations indicating hepatoid differentiation are related to the gene *CTNNB1,* which encodes β-catenin, and is known as being characteristic for the “non-proliferating class HCCs” (Sempoux et al. 2019; Maximin et al. 2014). In cHCC-ICC with predominance of the HCC component, alterations in *CTNNB1,* promoter of TERT *(pTERT)*, and *NFE2L2* seem to be predominant (Calderaro et al. 2023), as an auxiliary argument for the definition of HCC-type cHCC-ICC (Table 3).

Microsatellite instability (MSI-H) and the presence of mutations in the *KRAS, NRAS, BRAF, PTEN, ERBB2, FGFR2,* and *IDH1/2* genes reflect the biliary origins of cHCC-ICC (Roßner et al. 2023; Sempoux et al. 2019; Maximin et al. 2014; Calderaro et al. 2023). ICC does not show mutations for pTERT gene (Xue et al. 2019). Loss of heterozygosity (LOH) can be found at the same chromosomes as in HCC (1q, 71%; 8q, 57%; 8p, 57%) and ICC (3p, 6q) but also at 17p, 4q, 1p, 14q, 16q, 13q, 16p, and 9p (Roßner et al. 2023; Maximin et al. 2014).

Other genes that proved to be mutated in cHCC-ICCs are *AXIN1, RB1, PTEN, ARID2, BDR7ADGRV1, MUC2, NEB, DST,* and *HMCN1.* Epigenetic dysregulation was found in 19.5% of the cases and involved the genes *ARID1A, ARID2, PBRM1,* and *BRD7.* Cellular energetic disruption can be seen in 21.2% of the cases and is induced by mutations in the genes *KEAP1, IDH1, APOB,* and *ALB* (Xue et al. 2019).

Being a trigger of hepatocarcinogenesis, the *TP53* gene is mutated in 49%, 31%, and 22% of cHCC-ICC, pure HCC (known as “proliferating-class HCC”) and pure ICC, respectively (Xue et al. 2019). Hepatitis B infection might induce the inactivation of the *TP53* gene (Holczbauer et al. 2021).

In sarcomatoid cHCC-ICC, mutations in the *TP53* and *pTERT* genes were recently described, along with PDL-1 expression, as indicators of possible targeted therapy with immune checkpoint inhibitors (ICPIs) (Yoshuantari et al. 2023; Zheng et al. 2020).

## Prognosis, therapy, and survival

For patients with cHCC-ICC, the reported 5-year OS is 10–11%, like that of patients with pure HCC but better than that of patients with ICC (OS below 6%) (Garancini et al. 2014). A better OS rate was reported in the Asian population (Garancini et al. 2014; Lee et al. 2006, 2013; Yin et al. 2012), but the median survival time was 9–47 months only (Table 1).

The most common negative prognostic factors are a tumor size over 5 cm, the absence of capsule formation, vascular invasion, multiplicity, intrahepatic metastasis, TNM stage, distant spread, incomplete resection with positive margins or diagnosis in the non-operable phase, a high CA19-9 and/or AFP serum level and nestin positivity (Garancini et al. 2014; Lee et al. 2006; Yin et al. 2012; Zhang et al. 2024; Xue et al. 2019; Zhan et al. 2012).

Although the presence of lymph node metastases is also a negative factor and is significantly more frequent in cHCC-ICC cases than in patients with HCC (Table 2), the impact of lymphatic metastases on the 5-year OS is controversial (Garancini et al. 2014), like that of pre-existing disorders of the liver parenchyma (Steiner and Higgison 1959). Although cHCC-ICC is considered a variant of HCC, the ICC component can dictate the prognosis, especially because lymph node metastases mostly comprise an ICC architecture and immunoprofile (Lee et al. 2013; Zhao et al. 2016; Calderaro et al. 2023; Zhan et al. 2012). The reported median survival, in patients with cHCC-ICC and predominance of ICC component, is 29 months only (Calderaro et al. 2023). Besides liver recurrence, metastasis has been reported with a predilection for the lung (12%) and spine (6%) (Lee et al. 2006). HCC elements are mainly identified in microvascular invasion cells (Zhao et al. 2016) (Table 4).

**Table 4:** Effects of checkpoint inhibition of combined hepatocellular-cholangiocarcinoma (cHCC-ICC) based on the available 7 reports involving 27 patients (Satake et al. 2023; Saito et al. 2022; Rizell et al. 2020; Tahover 2019; Gigante et al. 2023; Zhou et al. 2023; Unome et al. 2024)

Author/year of publication	Age, gender, underlying diseases	Tumor biomarkers	Tumor characteristics	Immunohistochemistry and molecular features	Therapy	Side effects of immunotherapy	Time of immunotherapy	Clinical response
Patient 1 (Tahover 2019)	67, male	Alfa fetoprotein (AFP) and CA 19-9 normal, CA 125 210 U/mL (reference 2–35 U/mL)	Inoperable—only biopsy from the liver mass was available	Microsatellite stable (MSS), low mutational burden	Doublet—ipilimumab and nivolumab, followed by nivolumab	Hypothyroidism and Addison disease	11 months	Near complete response at PET-CT: CA125 decreased from 210 to 21 U/mL, ECOG performance status improved from 3 to 0
Patient 2 (Rizell et al. 2020)	53, female	NA	A 27 cm-sized tumor mass and two satellite lesions (25 and 30 mm); pulmonary metastases, no lymph node metastases; poorly differentiated tumor	Positive for arginase 1, AFP, glypican 3, cytokeratin 7/19, MUC1, Ki67 index 20%, negative for PD-L 1 (22C3 pharDx, Dako/Agilent), MSS	Sorafenib, followed by gemcitabine/cisplatin, then pembrolizumab as third line therapy	Sorafenib, followed by gemcitabine/cisplatin, then pembrolizumab as third line therapy Non-hemorrhagic diarrhea, leg edema, mild hypoalbuminemia, and immune-related hepatitis	9 months	Radiological complete remission of the three pulmonary metastases, AFP decreased from 1790 μg/L up to normal at 4 months after pembrolizumab, progression-free survival at 33 months after the start of checkpoint inhibition therapy
Patient 3 (Satake et al. 2023)	81, male, alcoholic liver disease	Normal	A 28 mm-sized unresectable tumor with multiple lymph node metastases; trabecular-sinusoidal architecture and glandular structures	Positive for HepPar-1, cytokeratin 19 and CD56, negative for PD-L1, MSS	Titanium silicate (TS)-1 for 13 months, followed by gemcitabine/cisplatin, then lenvatinib for 4 months, then atezolizumab plus bevacizumab, followed by atezolizumab	Proteinuria and ascites—improved by diuretics	7.5 months	Stable disease after two cycles bevacizumab and atezolizumab, but progressive disease after the 10th cycle; progression-free survival at 7.5 months after the start of immune-combined therapy, then progressive disease but still alive at 15 months after initiation of systemic therapy
Patient 4 (Satake et al. 2023)	62, male, chronic hepatitis C (HCV)	AFP 27443 μg/L, CA 19-9 433.1 u/mL, CEA 7.6 ng/mL	Unresectable stage II tumor, with portal vein invasion, no metastases	Cytokeratin 19	Atezolizumab plus bevacizumab	Hypertension grade 3, eruption grade 2, skin ulcer grade 1, high liver enzymes grade 2	15 months (ongoing)	Partial response at 4 months after the start of therapy
Patient 5 (Satake et al. 2023)	73, female	Normal	Unresectable stage II tumor, multicentric, no metastases	Cytokeratin 7	Atezolizumab plus bevacizumab, followed by gemcitabine/cisplatin plus TS-1	Interstitial lung disease grade 1, epistaxis grade 1	4 months	Partial response at 2 months after the start of therapy with gradual tumor shrinkage
Patient 6 (Satake et al. 2023)	76, male, prostate cancer	AFP 221.3 μg/L, CA 19-9 42.9 u/mL	Unresectable tumor, with portal vein invasion, bone metastases, no lymph node metastases	Cytokeratin 19	Atezolizumab plus bevacizumab, followed by lenvatinib	Eruption grade 1	2 months	Stable disease for 2 months only, thoracic aortic dissection at 2 months later, and progressive disease
Patient 7 (Satake et al. 2023)	76, male, bladder cancer	AFP 13.6 μg/L, CA 19-9 99.3 u/mL	Unresectable stage III multiple liver tumors, with lymph node metastases	Cytokeratin 7/19	Atezolizumab plus bevacizumab	Proteinuria grade 2	6 months (ongoing)	Stable disease, AFP and PIVKA-II decreased at two months after treatment initiation
Patient 8 (Satake et al. 2023)	74, male, chronic hepatitis C and pure HCC treated with proton therapy four years and seven months before	AFP 732 μg/L, CA 19-9 33.3 u/mL	Unresectable stage II multicentric tumor, no metastases; poorly differentiated tumor with ductal component	Cytokeratin 7/19	Gemcitabine/cisplatin plus TS-1, followed by atezolizumab plus bevacizumab	Hypertension grade 2, eruption grade 1, proteinuria grade 2, cerebral infarction	5 months	Partial response, stable disease for 2 months only
Patient 9 (Satake et al. 2023)	74, male, fatty liver and follicular lymphoma	AFP 31.5 μg/L, CA 19-9 28 u/mL	Unresectable stage II multicentric tumor, no metastases; the main component was ICC	NA	Gemcitabine/cisplatin plus TS-1, followed by atezolizumab plus bevacizumab	Anorexia, immune-related acute lymphocytic myocarditis grade 4, ascites	1 month	Progressive disease—stable disease for 2 months only, liver failure and death at two weeks after the onset of myocarditis
Patient 10 (Zhou et al. 2023)	53, male, 20-year history of hepatitis B, no antiretroviral therapy. mother with liver cancer	Normal	A 28 mm-sized solitary mass, alcoholic liver disease, cirrhosis	Low mutational burden, MSS, negative for PD-L1, several gene mutations (PTEN, TERT, GNAQ, FAT2, ROS1, CTNNB1, ERBB4)	Capecitabine—8 cycles, followed by triple therapy with sintilimab, lenvatinib, and nab-paclitaxel (8 cycles), then lenvatinib and sintilimab	Neutropenia grade 2, leukopenia grade 1, peripheral neuropathy grade 1	14 months	Partial response—tumor shrinkage after two cycles
Patient 11 (Unome et al. 2024)	49, female, no medical history	AFP 102.6 ng/mL, CA 19-9 32.6 U/mL, CEA 1.7 ng/mL, PIVKA-II 30 mAU/mL	Unresectable stage II tumor, multicentricity, no metastases	Cytokeratin 7/19, AFP	Durvalumab plus tremelimumab	Maculopapular rash grade 3	2 months	Progressive disease—stable disease for 2 months only, then bone and lymph node metastases, ascites, pleural effusion, AFP and CA 19-9 levels increased to 934 ng/mL and 111 U/mL
16 cases- Patients no 12–27 (Gigante et al. 2023)	12 males and 5 females, with a median age of 63 years (range, 42–71 years); associated hepatitis B (38%), hepatitis C (25%), cirrhosis (69%), metabolic syndrome (44%)	AFP median value 59.50 ng/mL (range 1.40–380.65), CA 19-9 2 U/mL (range 0.23–3.13)	Unresectable or metastatic tumors (n = 10); biopsies (n = 10) or surgical samples (n = 6); alcoholic liver disease (38%); multicentricity (75%); macrovascular invasion (31%)	KRAS amplification, mutations for CDK4, FGFR2, and SMARCA4 genes	Atezolizumab plus bevacizumab—first line (n = 9), second line after sorafenib or gemcitabine (n = 5), third line (n = 1) after gemcitabine + cisplatin, thus 5-fluorouracil + oxaliplatin, or fifth line (n = 1) after sorafenib, gemcitabine + oxaliplatin, 5-fluorouracil + irinotecan, and paclitaxel	Severe digestive bleeding (11%), ascites, anorexia grade 1 (33%), asthenia grade 1 and 2 (22%), proteinuria grade 2 (11%), thrombocytopenia (11%), polymyalgia rheumatic (n = 1), inappropriate antidiuretic hormone secretion (n- = 1)	6.4 months median time (range 1.3–31)	First line therapy—radiological progression (n = 4), partial response (n = 4), or stable disease (n = 1); second line therapy—stable disease (n = 4), partial response (n = 1); third line therapy (n = 1)—progressive disease, death at 3.9 months; Fifth line therapy (n = 1)—partial response after 15.5 months then stable disease for other 22 months

Besides the TNM stage, the OS is strongly related to the type of treatment (Table 1). It consists on local ablation with percutaneous ethanol injection or radiofrequency (RFA) and transarterial chemoembolization (TACE) or palliative supportive therapy versus liver curative resection and transplantation with associated systemic chemotherapy (Garancini et al. 2014; Azizi et al. 2020; Lee et al. 2013; Yin et al. 2012; Suciu et al. 2015). The presence of stem/progenitor cells is an indicator of resistance to therapy (Sempoux et al. 2019).

As cHCC-ICC is considered to have a biological potential that is intermediate between HCC and ICC (Sheng et al. 2023), most patients are still treated using the same protocols as for HCC (Chen et al. 2021), although the WHO includes cHCC-ICC in the group of hepatobiliary cancers (Sempoux et al. 2019).

RFA is recommended for patients with a maximum of three tumor nodules, confined to the liver, with none larger than 3 cm (Yin et al. 2012). Like for HCC or unresectable metastatic lesions of the liver, TACE is indicated for tumors larger than 3 cm, for patients with over three nodules, and for those with recurrent unresectable cHCC-ICC (Yin et al. 2012; Suciu et al. 2015) (Table 1). It is difficult to select patients who can respond to TACE because the occurrence of cHCC-ICC after TACE has also been reported (Sempoux et al. 2019). Moreover, as cHCC-ICC is less vascularized than pure HCC, it responds poorly to TACE (Yin et al. 2012; Chen et al. 2021; Fodor et al. 2019). Like HCC and ICC, COX-2 might be upregulated in cHCC-ICC, a factor that indicates a possibility for answering at anti-COX-2 therapy (Roßner et al. 2023; Suciu et al. 2015; Ishii et al. 2013).

Liver resection or transplantation is the optimal treatment for cHCC-ICC (Yin et al. 2012; Chen et al. 2021). Curative resection is defined by a post-operative cut surface free of cancer based on histologic assessment, obtained in patients with a maximum of three tumor nodules, without tumor thrombi in the portal vein or its branches, hepatic veins, or bile ducts, and without distant metastasis (Yin et al. 2012). This procedure can be successfully performed in about 40% of patients (Lee et al. 2013).

Liver transplantation significantly improved the prognosis and increased the 5-year OS to 40–48%, 68%, and 29% for patients with cHCC-ICC, pure HCC, and ICC, respectively (Garancini et al. 2014; Azizi et al. 2020). As patients with non-HCC tumors of the liver are not considered eligible for transplantation, it is difficult to assess the efficacy of such therapy. In most patients, the preliminary diagnosis was HCC, probably due to the examination of a small biopsy specimen only. If a reclassification of cHCC-ICC would be done, donor organs can be prioritized for patients with chances for longer survival rate (Calderaro et al. 2023).

Supportive and palliative therapy is administered in about 10% and 47% of cases, respectively (Lee et al. 2013). It is suitable for patients with an advanced-stage cHCC-ICC diagnosis, with vein thrombosis or ascites, extrahepatic tumor spread, unresectable tumors, high serum level of hepatic enzymes, and compromised liver function (Yin et al. 2012; Zhang et al. 2024).

Due to rarity, heterogeneity and complexity, standard first-line systemic therapy for patients diagnosed with cHCC-ICC is lacking (Zhang et al. 2024; Xu et al. 2023; Trikalinos et al. 2018). Gemcitabine or capecitabine and platinum-based agents (cisplatin or oxaliplatin) are used in most cases and proved the most beneficial therapy (Azizi et al. 2020; Zhang et al. 2024; Xu et al. 2023; Trikalinos et al. 2018; Salimon et al. 2018). As patients can also have associated cirrhosis and compromised liver function (Table 4), the minimum dose or hepatic arterial infusion is indicated (Azizi et al. 2020; Zhang et al. 2024; Trikalinos et al. 2018; Salimon et al. 2018).

Other therapeutic options include tirozinkinases such as sorafenib (Table 1), lentiviruses, 5-fluorouracil, and antiangiogenic drugs such as bevacizumab but results are under expectations (Azizi et al. 2020; Zhang et al. 2024) (Table 4). Sorafenib proved to have a better efficacy if HCC was predominant (Fig. 2) (Zhang et al. 2024). As *CTNNB1*-mutated cases proved to show an immune-high profile (Nguyen et al. 2022) and these cases are mostly HCC-type cHCC-ICCs, they might be resistant at ICPIs (Akiba et al. 2024).

As the ICC component can show MSI-H status and PDL-1 is highly expressed in sarcomatoid cHCC-ICC (Yoshuantari et al. 2023), it is thought that promising results can be obtained with ICPIs (Fig. 2). Good efficacy and quality of life were recently reported among patients who were administered a second-line treatment of durvalumab or lenvatinib plus programmed death-1 (PD-1) antibody (Zhan et al. 2012; Oh et al. 2022).

Data on the efficacy of ICPIs in the therapy of cHCC-ICC are scarce and cHCC-ICC are considered ineligible for most of the clinical trials (Yau et al. 2024). Only seven articles involving 27 patients were published and reported the therapeutic efficacy of ICPIs in unresectable, inoperable, or metastatic cHCC-ICC (Satake et al. 2023; Saito et al. 2022; Rizell et al. 2020; Tahover 2019; Gigante et al. 2023; Zhou et al. 2023; Unome et al. 2024) (Table 4). Due to the rarity of cHCC-ICC, it is difficult to organize prospective clinical studies. Therefore, phase 2 or 3 clinical trials are not available for cHCC-ICC (Gigante et al. 2023; Pinter et al. 2023).

## Summary and future perspectives

The available data from the literature highlight that cHCC-ICC is an intermediate between HCC and ICC in terms of characteristics, behavior, and prognosis. Based on the predominance of differentiation of pluripotent cells, cHCC-ICC should be classified as HCC-type cHCC-ICC and ICC-type cHCC-ICC. As liver transplant improves survival in only one-quarter of patients with cHCC-ICC, patients with HCC-type cHCC-ICC should be considered at risk for microvascular invasion and treated similarly to those with HCC. On the other hand, in patients with ICC-type cHCC-ICC as per histological assessment and immunoprofile, like in patients with lymph node metastases comprising ICC architecture, the therapeutic approach needs to be more aggressive and use strategies that are preferred for ICC. Large cohorts need to be examined to check the true incidence of the two proposed subtypes of cHCC-ICC and the impact of lymph node metastases-focused therapy in further oncologic management.

## Data Availability

The literature data used to support the findings of this study are available from the corresponding author upon request. This is a systematic review of literature.
